# Assessing the Genome-Wide Effect of Promoter Region Tandem Repeat Natural Variation on Gene Expression

**DOI:** 10.1534/g3.112.004663

**Published:** 2012-12-01

**Authors:** Martha H. Elmore, John G. Gibbons, Antonis Rokas

**Affiliations:** Department of Biological Sciences, Vanderbilt University, Nashville, Tennessee 37235

**Keywords:** molecular phenotype, genotype−phenotype map, tandem repeat copy number polymorphism, RNA-Seq

## Abstract

Copy number polymorphisms of nucleotide tandem repeat (TR) regions, such as microsatellites and minisatellites, are mutationally reversible and highly abundant in eukaryotic genomes. Studies linking TR polymorphism to phenotypic variation have led some to suggest that TR variation modulates and majorly contributes to phenotypic variation; however, studies in which the authors assess the genome-wide impact of TR variation on phenotype are lacking. To address this question, we quantified relationships between polymorphism levels in 143 genome-wide promoter region TRs across 16 isolates of the filamentous fungus *Aspergillus flavus* and its ecotype *Aspergillus oryzae* with expression levels of their downstream genes. We found that only 4.3% of relationships tested were significant; these findings were consistent with models in which TRs act as “tuning,” “volume,” or “optimality” “knobs” of phenotype but not with “switch” models. Furthermore, the promoter regions of differentially expressed genes between *A. oryzae* and *A. flavus* did not show TR enrichment, suggesting that genome-wide differences in molecular phenotype between the two species are not significantly associated with TRs. Although in some cases TR polymorphisms do contribute to transcript abundance variation, these results argue that at least in this case, TRs might not be major modulators of variation in phenotype.

Nucleotide tandem repeats (TRs) are ubiquitous in eukaryotic genomes and occur in both coding and noncoding regions (Li *et al.* 2002). TRs consisting of short repeat units or copies made up of 1–9 base pairs typically are defined as microsatellites, whereas TRs whose repeat units are much longer are called minisatellites. Changes in TR copy number occur 100 to 10,000 times more often than point mutations (Lynch *et al.* 2008), and unlike standard mutations, they often are reversible (Kashi and King 2006).

Because of their abundance, high variability, and presumed selective neutrality, TR copy number polymorphisms (TRCNPs) have been used extensively in evolutionary genetics and epidemiological studies (Goldstein and Schlötterer 1999). However, TRCNPs also have been shown to directly alter phenotype in both coding (Sawyer *et al.* 1997; Fondon and Garner 2004; Verstrepen *et al.* 2005) and noncoding regions (Hammock *et al.* 2005; Rockman *et al.* 2005; Vinces *et al.* 2009). For example, TRCNPs in different protein-coding genes in birds (Johnsen *et al.* 2007), fruit flies (Sawyer *et al.* 1997), and filamentous fungi (Michael *et al.* 2007) are directly involved in fine-tuning circadian rhythm periodicity, whereas TRCNPs in *cis*-regulatory regions have been linked to gene expression level modulation in humans (Bennett *et al.* 1995), voles (Hammock and Young 2004), fish (Streelman and Kocher 2002), and fungi (Staib *et al.* 2002; Vinces *et al.* 2009). In one of the best documented examples, experimental manipulation of TR copy number in the promoter of the *MET3* and *SDT1* genes in the baker’s yeast *Saccharomyces cerevisiae* altered gene expression in a bell curve−like pattern; expression was relatively low at small TR copy numbers, increased at intermediate copy numbers, and was reduced again as TR copy numbers increased further (Vinces *et al.* 2009).

The abundant, continuous, and potentially reversible mutational variation offered by TRCNPs coupled with their demonstrated involvement in modulating phenotype in several case studies has led to the hypothesis that TRs act as “evolutionary knobs” of molecular (*e.g.*, gene expression) and organismal (*e.g.*, circadian rhythm periodicity) phenotypes (Kashi *et al.* 1997; King *et al.* 1997; Sawyer *et al.* 1997; Fondon and Garner 2004; Hammock and Young 2004; Kashi and King 2006; Fondon *et al.* 2008; Vinces *et al.* 2009). Because of the complexity of the genotype–phenotype map, in principle TRCNPs can affect phenotype in a variety of different ways. Nevertheless, experimental data argue that the relationship between TRCNPs and phenotype fits into a few general patterns; the “volume knob,” “tuning knob,” “optimality knob,” and “switch” patterns. In the “volume knob” pattern, TR copy number is negatively or positively associated with phenotype (Schilling *et al.* 1995; Sawyer *et al.* 1997; Verstrepen *et al.* 2005), whereas in the “tuning knob” pattern, changes in copy number, much like a radio tuning dial, cause corresponding nonlinear changes to phenotype (Vinces *et al.* 2009). In contrast, in the “optimality knob” pattern gene expression increases up to a point and then decreases as copy number increases further (or vice versa). Finally, TRCNPs also can act as switches, turning on and off, or vastly changing, phenotypes when copy number crosses a particular threshold (Chen *et al.* 2010).

Although the potential importance of TRs in noncoding regions is evident, studies that evaluate their effect on gene activity on a genome-wide level, and that explicitly test the hypothesis that they are major contributors to phenotypic variation, are lacking (Vinces *et al.* 2009). To gauge the impact of TRCNPs on gene activity, we measured TR allele length across 143 promoter regions in 16 isolates of the closely related fungal species *Aspergillus flavus* and its domesticated relative *Aspergillus oryzae* (Gibbons *et al.* 2012b) and for every locus compared the degree of TR polymorphism to the level of gene expression. Although our results identified several genes whose expression correlated with promoter region TR copy number in a manner consistent with the “tuning knob,” “volume knob,” and “optimality” patterns, the majority of the 143 loci we examined did not show a significant relationship between promoter region TRCNPs and gene expression. Moreover, we found no evidence that genes whose promoter regions contain TRs exhibit greater expression variance or that the promoter regions of genes that are significantly differentially expressed between the two species are enriched in TRs. Although it is abundantly clear that TRCNPs do contribute to molecular phenotype, these results argue that TR variation might explain only a small fraction of the variation in phenotype observed within and between *A. oryzae* and *A. flavus*.

## Materials and Methods

### Identification of TRs in promoter regions

**Figure 1:**
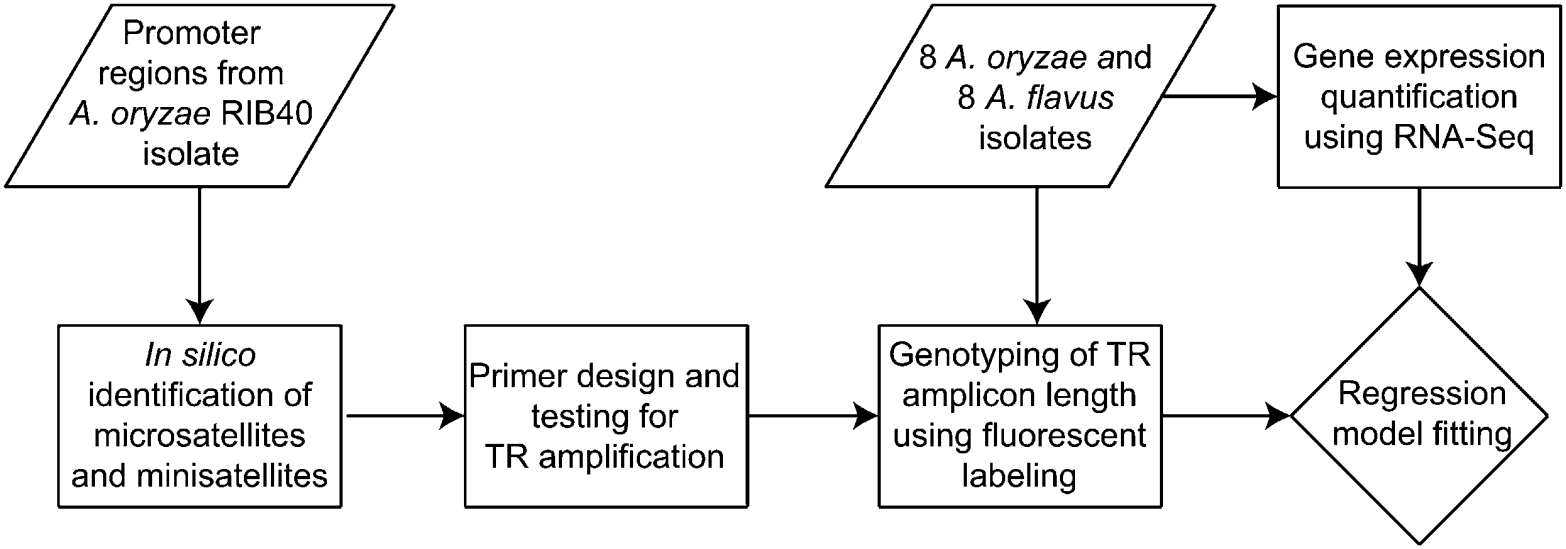
Design of the experiment to test the genome-wide effect of TR variation in modulating molecular phenotype.

Our experimental design is depicted in Figure 1. We defined promoter regions as the noncoding 1000 bp region upstream of annotated start codons. The EMBOSS etandem software (Rice *et al.* 2000) was used to identify TRs in the promoter regions of the *A. oryzae* RIB 40 reference genome (Machida *et al.* 2005). We validated the conservation of *A. oryzae* TRs in the *A. flavus* genome by checking for their presence in the corresponding orthologous region of the *A. flavus* reference strain NRRL 3357 genome. We defined microsatellites as sequence repeats between 2–9 bp and minisatellites as sequence repeats ≥10 bp.

We considered TRs as significant if the repeat unit consensus sequence conservation was ≥90%. We imposed this strict cutoff to ensure the number of imperfect TRs was small because we were particularly interested in analyzing polymorphic TRs, and previous work has shown that “pure tract” TRs have greater rates of polymorphism compared with “impure tract” TRs (Butland *et al.* 2007). In addition, it is important to note that by default the etandem software requires a minimum sequence length of 24 bp, which means that dinucleotide repeats were required to have at least 12 copies, trinucleotide repeats were required to have at least eight copies, and so on. Again, for our purposes, these parameters were favorable in the identification of polymorphic TRs because TR copy number is positively associated with elevated mutation rates (Anmarkrud *et al.* 2008).

### Culture of fungal isolates and nucleic acid extraction

We analyzed eight isolates of *A. oryzae* (isolate accession numbers RIB 333, RIB 642, RIB 331, RIB 302, RIB 40, RIB 537, RIB 632, and RIB 949) and eight isolates of *A. flavus* [isolate accession numbers SRRC 1357, SRRC 2112, SRRC 2632, SRRC 2524, SRRC 2653, SRRC 2114, SRRC 1273, and NRRL 3357 (Gibbons *et al.* 2012b)]. For genomic DNA extraction, spores were inoculated in potato dextrose broth and grown at room temperature in a tissue rotator; after 3 days’ growth, the mycelium was harvested and ground in liquid nitrogen. Genomic DNA was extracted using a standard CTAB protocol (Stewart and Via 1993).

For RNA extraction, 500 μL of a water conidial suspension (10^7^/mL) was spread onto a potato dextrose agar plate covered with a layer of sterile porous cellophane and grown at 30° for 24 hr. Mycelium was harvested with a metal spatula, flash frozen in liquid nitrogen, and stored at –80°. Mycelium was ground with a mortar and pestle in liquid nitrogen. Total RNA was extracted using TRIzol (Life Technologies), DNased, then cleaned with an RNeasy column (QIAGEN) according to the manufacturer’s instructions.

### Primer design

Primer pairs targeting TR-containing promoter regions were designed using the Primer3 software (Rozen and Skaletsky 2000). Forward and reverse primers were designed around target regions so that the expected amplicon size would be between 150–450 bp. We incorporated a dinucleotide GC clamp into primer pairs when possible and a M13 tag to the 5′ end of all forward primers for use in downstream fluorescent genotyping (Schuelke 2000).

### TR genotyping

We genotyped the 16 *A. oryzae* and *A. flavus* isolates across 143 TR loci (72 microsatellite and 71 minisatellite loci) using the fluorescent amplicon labeling approach described by Schuelke (Schuelke 2000). A touchdown PCR protocol (Don *et al.* 1991) was implemented to limit nonspecific amplification and consisted of the following cycling profile: 95° for 3 min, 11 cycles of 94° for 30 sec, 65° for 30 sec (with annealing temperature dropping 1° per cycle) and 72° for 45 sec, followed by 29 cycles of 94° for 30 sec, 53° for 30 sec, 72° for 45 sec, followed by a final extension of 72° for 20 min. PCR products were sized on an ABI 3730xl Genetic Analyzer at Genewiz (South Plainfield, NJ) using the LIZ500 size standard. Amplicon lengths were called using the Peak Scanner Software v1.0 (ABI). Due to the uncertainty in extracting repeat unit number directly from amplicon length (Guichoux *et al.* 2011) we used normalized allele length (NAL) to measure the repeat copy number for each allele at a TR locus:

NAL=amplicons length/length of the smallest amplicons at locus

### RNA-Seq

RNA-Seq libraries were constructed and sequenced at the Vanderbilt Genome Sciences Resource using the Illumina Tru-seq RNA sample prep kit as previously described (Gibbons *et al.* 2012a,b). In summary, total RNA quality was assessed via Bioanalyzer (Agilent). Upon passing quality control, poly-A RNA was purified from total RNA and second strand cDNA was synthesized from mRNA. cDNA ends were then blunt repaired and given a adenylated 3′ end. Next, barcoded adapters were ligated to the adenylated ends and the libraries were PCR enriched, quantified, pooled and sequenced an on Illumina HiSequation 2000 sequencer.

### Gene expression quantification

For each sample, Illumina generated mRNA reads were trimmed to 40 bp and independently mapped against the *A. oryzae* RIB40 reference transcriptome (Machida *et al.* 2005) allowing 2 mismatches per read. Gene expression levels were quantified in terms of reads per kilobase of transcript per million mapped reads (RPKM) (Mortazavi *et al.* 2008), a self-normalized value of absolute transcript abundance, by use of the rSeq package (Jiang and Wong 2009) as previously described (Gibbons *et al.* 2012a,b).

### TR representation in genes that are differentially expressed between species

If TRs are a major source of molecular phenotypic variation between populations or species, one would minimally expect TRs to be overrepresented in the promoter regions of genes that are significantly differentially expressed between them. To test this hypothesis, we compared the frequencies of promoter region TRs between the subset of *A. oryzae* and *A. flavus* differentially expressed and nondifferentially expressed genes using a Fisher’s exact test. The differentially expressed gene set was determined by comparing the difference in sample mean gene expression level (RPKM) of each gene between the 8 *A. oryzae* and 8 *A. flavus* isolates imposing a *t*-test *P*-value cutoff of 0.05 (904 total genes).

### Testing the hypothesis that TRs are “evolutionary knobs”

We investigated the relationship between promoter region TRCNP, as measured by NAL, and downstream gene expression, as measured by RPKM, at each locus for each species separately as well as combined, using a series of regression models chosen to represent biologically relevant patterns. The optimal or best-fit regression model for each locus was assessed by choosing the model with the smallest sample size corrected Akaike’s Information Criterion value:

$$AICc=\log_{e}\left(RSS\right)+2K+\frac{2K*\left(K+1\right)}{n-K-1}$$

where *RSS* is the regression residual sum of squares, *K* equals the number of parameters in the model, and *n* is the number of observations (Akaike 1974). Comparisons in which alleles were fixed (n = 28), or for which genes were not expressed in all samples (n = 9) were not analyzed (n = 392 total tests). For each optimal regression model, *P* values were calculated from the *F* ratio based on the *F* distribution (Sokal and Rohlf 1995), and results were reported using a significance threshold of *P* < 0.01 as well as a Bonferroni multiple test corrected *P*-value cutoff of 0.000128 (*P* value = 0.05/392 tests).

We also tested whether the relationship between promoter region TRCNP and downstream gene expression fit a “switch” model by examining for significant differences in expression levels between alleles of a given locus. Specifically, we compared the expression levels of loci harboring multiple alleles with frequencies ≥0.25 in *A. oryzae* by using either a *t*-test (for two alleles) or analysis of variance (for three alleles). Results were reported using a significance threshold cutoff of *P* < 0.01 as well as a Bonferroni multiple test corrected *P*-value cutoff of 0.00135.

### Testing the hypothesis that TRs contribute to gene expression “noise”

To test the hypothesis that TRs in promoter regions may increase expression noise, we evaluated whether genes containing TRs in their promoter regions had greater expression variance than two sets of background genes. In the first comparison, to control for biases due to genome location, we compared the set of TR-containing loci against a background set comprising genes lacking TRs in their respective promoters and that were located two genes upstream and two genes downstream of TR-containing loci. In the case of the microsatellite set, we analyzed the total TR gene set (n = 71), as well as only the polymorphic TR gene set (n = 66). No TR alleles were fixed in the minisatellite gene set. In the second comparison, to control for biases due to gene function, we compared the set of TR-containing loci against 10 different random background sets of genes that have the same functional classifications according to the FunCat Annotation Database (Ruepp *et al.* 2004). In cases in which a single gene was classified in more than one FunCat category, we randomly assigned it to one of them. We tested the statistical significance of all comparisons using Tukey-Kramer post-hoc analysis of variance tests. No TR alleles were fixed in the FunCat gene set. All statistical analyses were performed in JMP version 9 (http://www.jmp.com/).

## Results

### Distribution and genotyping of promoter region TRs

We identified 228 TRs in 190 promoter regions of the *A. oryzae* RIB 40 reference genome (Machida *et al.* 2005), several of which contained both microsatellites and minisatellites. Specifically, 127 microsatellites and 101 minisatellites were identified in 125 and 99 promoter regions, respectively. A total of 57% (72/127) and 70% (71/101) of these microsatellite and minisatellite containing regions were successfully genotyped, respectively (Supporting Information, Table S1).

### Patterns of TR allele length and expression variance

From the 72 promoter region microsatellites and 71 minisatellites, 11 and 6 microsatellite loci and 6 and 1 minisatellite loci showed no variation in *A. oryzae* and *A. flavus*, respectively. Of the 72 microsatellite loci, 18 had significantly reduced allelic variance in *A. oryzae*, and only three loci had reduced variance in *A. flavus* (*F*-test; *P* < 0.0007). Of the 71 minisatellite loci, 11 and 9 had reduced allelic variance in *A. oryzae* and *A. flavus*, respectively (*F*-test; *P* < 0.0007; Table S2). The reduction of allelic variation in *A. oryzae* mirrors the overall reduction of genetic variation in these isolates compared with *A. flavus* (Gibbons *et al.* 2012b). Of the 123 genes for which we analyzed gene expression (several genes had multiple TRs in their promoter), 4 and 7 had reduced expression variance in *A. oryzae* and *A. flavus*, respectively (*F*-test; *P* < 0.0004; Table S3).

### TRs are not overrepresented in the promoter regions of differentially expressed genes

If TRCNP in *cis*-regulatory regions is a major source of rapid phenotypic evolution, we would expect that genes that are significantly differentially expressed between populations or species to contain more TRs in their promoter regions than background genes. We identified 904 differentially expressed genes between *A. oryzae* and *A. flavus* (see *Materials and Methods*). Comparison of the frequencies of promoter region TRs in differentially expressed and nondifferentially expressed genes between *A. oryzae* and *A. flavus* showed that 1.33% (12 differentially expressed genes of 127 genes containing upstream TRs) and 1.03% (892 differentially expressed genes of 11,936 genes lacking upstream TRs) of differentially expressed and nondifferentially expressed genes, respectively, contained promoter region TRs. TRs were not significantly overrepresented in the differentially expressed gene set (Fisher exact test; *P* = 0.39).

### Polymorphism in promoter region TRs is infrequently associated with modulation of gene expression

**Figure 2:**
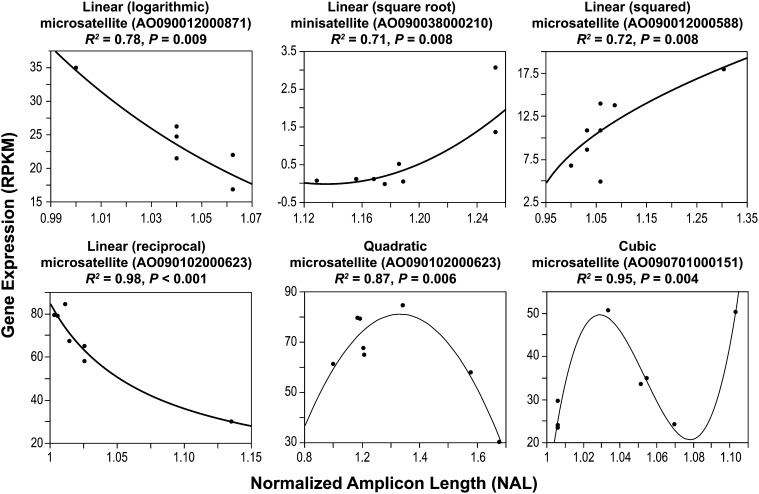
Select patterns of significant relationships between promoter region TR polymorphism and variation in gene expression in *A. flavus*. For each example, the regression fit, TR type (microsatellite or minisatellite), locus identifier (in parentheses), regression *R^2^*, and regression *P* values are provided. Each plot shows the NAL of TR on the x-axis and gene expression level (RPKM) on the y-axis.

We investigated the relationship between promoter region TR allele length (NAL; Table S2) and gene expression (RPKM; Table S3) both by species and combined, using a series of regression models chosen to represent biologically relevant patterns (Figure 2). Specifically, we chose a series of *untransformed* and *transformed* (*logarithmic*, *square root*, *squared*, and *reciprocal*) *linear* regression models because they correspond to “volume knob” patterns in which TR copy number changes directly correlate with gene expression levels. We chose the *quadratic* model because it corresponds to the “optimality knob” pattern in which gene expression increases (or decreases) up to a point and then decreases (or increases) as copy number increases further. Finally, we chose the *cubic* model because it resembles the “tuning knob” pattern in which gene expression oscillates in step with TR variation.

In total, only 4.3% (17 of 392) of comparisons showed a significant relationship (Table 1) at the *P* < 0.01 level (only three comparisons withstood the Bonferroni corrected *P* < 0.000128). We identified 6, 1, and 2 significant relationships within the microsatellite loci and 3, 3 and 2 significant relationships within the minisatellite loci in the *A. flavus*, *A. oryzae*, and the combined data, respectively. These relationships fit several different regression models (1 *untransformed linear*, 4 *transformed linear*, 3 *quadratic*, and 8 *cubic*; Figure 2). We found no evidence that the 17 TRs with significant relationships were more likely to be located in closer proximity to the start codon. Specifically, these TRs were not preferentially found within 100 bp, 150 bp, 200 bp or 500 bp upstream of the start codon (Fisher exact test; *P* ≥ 0.60 for all comparisons). Finally, comparison of the expression values of 49 genes with promoter region TR alleles (12 microsatellite and 37 minisatellite) occurring at high frequencies showed no significant differences in the expression levels of different alleles in any of our comparisons (at the *P* < 0.01 level), suggesting that none of these TRCNPs likely function as expression “switches.”

**Table 1:** Loci in which promoter region TR variation correlates significantly with gene expression variation

Type	Species	Gene Promoter	Function	Best-Fit Regression	R^2^	*P* value
Microsatellite	*A. flavus*	AO090012000588	SNF2 family helicase/ATPase	Linear (squared)	0.72	0.008
		AO090012000871	PAP2 superfamily	Linear (logarithmic)	0.78	0.009
		AO090102000623	HLH transcription factor	Quadratic	0.87	0.006
		AO090206000041	F-box domain	Cubic	0.94	0.007
		AO090701000151	Growth-arrest-specific protein 2 domain	Cubic	0.95	0.004
		AO090701000375	RhoGAP domain	Quadratic	0.96	0.002
	*A. oryzae*	AO090005000013	Uncharacterized protein	Cubic	0.96	1.00E-04
	combined	AO090003000121	6-phosphogluconate dehydrogenase	Cubic	0.67	0.009
		AO090012000871	PAP2 domain-containing protein	Cubic	0.64	0.009
Minisatellite	*A. flavus*	AO090005000959	Hypothetical protein	Linear (reciprocal)	0.83	0.002
		AO090038000210	Polyketide synthase	Linear (square root)	0.71	0.008
		AO090102000623	HLH transcription factor	Linear (reciprocal)	0.98	1.55E-05
	*A. oryzae*	AO090005000567	Hypothetical protein	Cubic	1.00	2.77E-27
		AO090009000040	Hypothetical protein	Cubic	0.98	0.001
		AO090010000582	Eukaryotic-type carbonic anhydrase	Cubic	0.95	0.004
	combined	AO090005000567	Hypothetical protein	Quadratic	0.74	2.00E-04
		AO090102000623	HLH transcription factor	Linear (untransformed)	0.46	0.005

TR, tandem repeats.

### Promoter-region TRs do not generate expression noise

**Figure 3:**
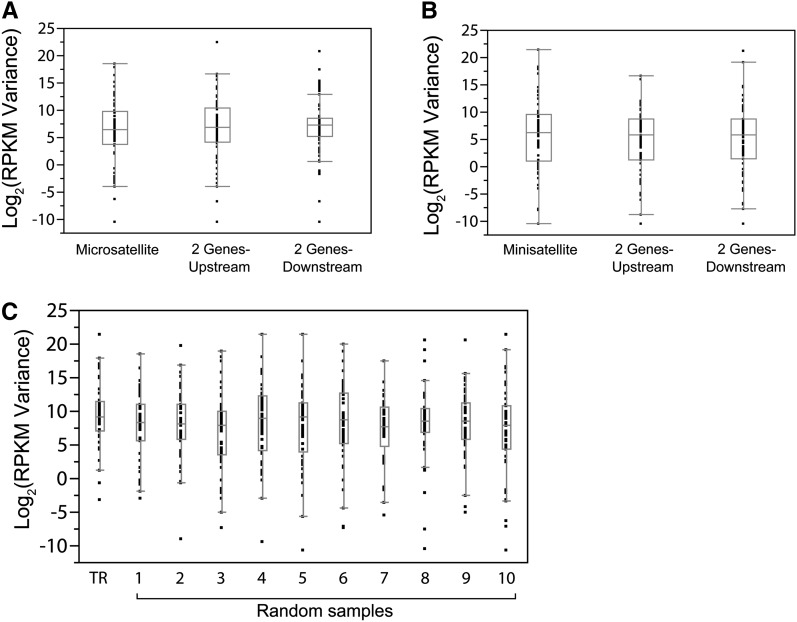
Gene expression variance is not increased in genes with promoter region TRs. Expression variance comparison between genes containing promoter region TRs and those without promoter region TRs but are located in the same genomic region (A and B) or have similar functions (C). For each box plot, the horizontal line represents the sample median, the box extends from the first to the third quartile, and the whiskers extend to the interquartile ranges. For (C), “TR” represents the TR dataset (combined microsatellite and minisatellite), whereas numbers 1–10 represent each of the randomly selected sets of genes with similar functional classifications lacking promoter region TRs.

Even if the relationship between promoter-region TRCNP and downstream gene expression at a given locus does not fit a particular model, because TR mutational variation is abundant, continuous, and potentially reversible, one might expect that the expression of genes containing TRCNPs in their promoter regions might be more “noisy” relative to background genes. We tested this hypothesis by comparing the distributions of expression variance between genes containing promoter region TRs against background sets of genes lacking promoter region TRs that are either from the same genomic region or have the same functional annotation. We found no significant difference in the expression variation between genes with promoter region TRs and background genes from the same genomic region (Tukey Kramer; microsatellite: *vs.* up-stream *P =* 0.93, *vs.* down-stream *P =* 0.62 and minisatellite: *vs.* up-stream *P =* 0.52, *vs.* down-stream *P =* 0.75; Figure 3, A and B) or with the same functional annotation (Tukey Kramer; all *P* > 0.65; Figure 3C), arguing that promoter region TRs are not acting as expression “noise makers.” For the microsatellite data set, similar results were obtained when only the polymorphic TR gene set was analyzed (Tukey Kramer; microsatellite: *vs.* up-stream *P =* 0.97, *vs.* down-stream *P =* 0.85).

Conversely, rather than generate phenotypic variability (*e.g.*, expression noise), some TRs may in fact function to stabilize or repress variation in gene expression. The presence, and abundance, of TRs is a staple of heterochromatin, the condensed and transcriptionally inactive segments of chromosomal DNA. If TRs are playing a critical role in the repression of gene expression by promoting heterochromatin formation, we would expect to see no expression of many genes with upstream TRs. However, only 1 of the 127 unique genes containing upstream TRs was not expressed in all 16 isolates, which was not statistically different from the number of genes that were not expressed in genes lacking upstream TRs (133 of 11,803 genes; Fisher exact test; *P* = 1.00).

## Discussion

Decades of work in a variety of organisms and study of a variety of phenotypes have provided abundant evidence that specific TRCNPs in both coding and noncoding regions alter both molecular and morphological phenotypes (Bennett *et al.* 1995; Sawyer *et al.* 1997; Staib *et al.* 2002; Streelman and Kocher 2002; Fondon and Garner 2004; Hammock and Young 2004; Hammock *et al.* 2005; Rockman *et al.* 2005; Verstrepen *et al.* 2005; Johnsen *et al.* 2007; Michael *et al.* 2007; Vinces *et al.* 2009). Although our examination of the genome-wide effect of promoter region TRCNPs on gene expression in the close relatives *A. flavus* and *A. oryzae* is in agreement with these studies, it did not provide support for the hypothesis that TRCNPs generally act as “evolutionary knobs” or “switches” of molecular phenotype [Table 1, Figures 2 and 3 (King *et al.* 1997; Fondon and Garner 2004; Kashi and King 2006; Fondon *et al.* 2008)]. One complicating factor in our study is that we evaluated the relationship between TRCNPs and molecular phenotype in only one growth condition. However, examination of gene expression of three of the *A. oryza*e and three of the A. *flavus* isolates when grown on rice also does not support a significant link between TRs and molecular phenotype (Gibbons *et al.* 2012b). Furthermore, although our study used a small number of samples, we note that other studies have shown significant relationships between TRCNP and gene expression using similar numbers of data points (Vinces *et al.* 2009). Although the number of significant relationships could potentially increase if more samples were examined, the requirement of larger sample sizes to detect a phenotypic effect would further support our argument that the association between phenotypic and TR variation is weak at best.

TRs have orders of magnitude higher mutation rates than point mutations and TR allele states are reversible, two attributes that are often viewed as advantageous when TRs are compared against other standard sources of genetic variation that contribute to evolution (King *et al.* 1997). However, a well-established and supported tenet of evolutionary theory is that, because most new mutations are deleterious, selection in all organisms will act to reduce mutation rate toward the physiology- or selection-imposed minimum (Lynch 2010). Thus, in principle it is unlikely that a type of variation with high mutational instability, like TRs, would be a major contributor to phenotypic evolution. Support for this argument is provided by the knowledge that TRs are not evolutionarily stable features of eukaryotic genomes (Huntley and Clark 2007; Gibbons and Rokas 2009), as well as by dozens of human genetic diseases, which suggest that a significant fraction of the variation present in TRCNPs is deleterious (Mirkin 2007). These caveats notwithstanding, the mutational instability of TRCNPs might be beneficial in certain specific cases, such as in cell-surface genes from organisms that live in rapidly fluctuating environments (Verstrepen *et al.* 2005; Vinces *et al.* 2009).

Two of the largest screens of the effect of TR variation on phenotype have been the examination of 37 TRs in 17 dog genes (Fondon and Garner 2004), and the examination of 33 random TRs in promoter regions of an equivalent number of yeast genes (Vinces *et al.* 2009). Although both studies provide strong evidence that specific TRs modulate molecular and morphological phenotype, certain aspects of both study systems are not representative of other organisms. For example, whereas 25% of yeast promoters contain one or more TRs (Vinces *et al.* 2009), this is true of only 1.5% (190/12,603) of *A. oryzae* promoters. Similarly, the very strong artificial selection imposed on domesticated dog breeds, which has resulted in the accumulation of large numbers of deleterious mutations (Cruz *et al.* 2008), suggests that their genetic architecture is unlikely to be representative of that of genomes that have been sculpted by natural selection in the wild.

Perhaps more importantly, the question asked by these, as well as by all other studies that have so far examined the relationship between TRCNPs and molecular phenotype is “whether TRCNPs are significantly associated with phenotype.” We believe that this question is orthogonal to the question of interest, namely, “whether differences in phenotype are significantly associated with TRCNPs”, because it is this second question that specifically addresses whether TRs are majorly contributing to phenotypic differences within and between species. If TRs played an important role in supplying rapid phenotypic variation to populations experiencing unique selective pressures, such as ones undergoing domestication (Fondon and Garner 2004), we would expect to observe that differences in molecular phenotype between species are significantly enriched with TR-containing loci. However, only 1.3% of the genes that are significantly differentially expressed between the domesticated filamentous fungus *A. oryzae* and its wild relative *A. flavus* (Gibbons *et al.* 2012b) contained TRs in their promoter regions, a percentage not significantly different from the background, arguing that differences in molecular phenotype between the two species are largely explained by molecular variation unrelated to TRCNPs. In conclusion, although some TRCNPs do contribute to phenotype, both experimental data and theoretical considerations indicate that they might not always be a predominant source of genetic variation in phenotypic evolution.

## Supplementary Material

Supporting Information
