# Supplementary material for: Assessing the Genome-Wide Effect of Promoter Region Tandem Repeat Natural Variation on Gene Expression
Source: G3 (Bethesda). 2012 Dec 1;2(12):1643–9. doi: 10.1534/g3.112.004663 (PMC3516485; doi:10.1534/g3.112.004663)
Supplement: Supporting Information [file supp_2_12_1643__index.html]

Supporting Information 

# Assessing the Genome-Wide Effect of Promoter Region Tandem Repeat Natural Variation on Gene Expression

## Supporting Information for Elmore *et al.*, 2012

**Files in this Data Supplement:**

- Table S1 - Characteristics of TRs and Primers (.xlsx, 24 KB)
- Table S2 - Microsatellite Fragment length (Bp) of amplified loci for each of the 16 isolates (.xlsx, 45 KB)
- Table S3 - Gene expression (RPKM) for microsatellite loci (.xlsx, 35 KB)
